# Pacific Med. and Surg. Journal

**Published:** 1859-03

**Authors:** 


*Pacific Med. and Surg. Journal.*—We have missed the last few numbers of this Journal, and, as we see extracts from it in some of our exchanges, conclude that it has not met with the fate of its predecessors in California, but has forgotten our existence. Will the editors please remember us.

